# Marine ecosystem health status assessment through integrative biomarker indices: a comparative study after the Prestige oil spill “Mussel Watch”

**DOI:** 10.1007/s10646-013-1042-4

**Published:** 2013-02-23

**Authors:** Ionan Marigómez, Larraitz Garmendia, Manu Soto, Amaia Orbea, Urtzi Izagirre, Miren P. Cajaraville

**Affiliations:** CBET Ikerketa-Taldea, Zoologia eta Biologia Zelularra Saila, Plentziako Itsas Estazioa (PIE), Universidad del País Vasco/Euskal Herriko Unibertsitatea, Areatza Z/G, 48620 Plentzia-Bizkaia, Basque Country Spain

**Keywords:** Ecosystem health assessment, Mussels, Prestige oil spill, Integrative indices, Biomarkers

## Abstract

Five integrative biomarker indices are compared: Bioeffects Assessment Index (BAI), Health Status Index (HSI), integrated biological response (IBR), ecosystem health condition chart (EHCC) and Integrative Biomarker Index (IBI). They were calculated on the basis of selected biomarker data collected in the framework of the Prestige oil spill (POS) Mussel Watch monitoring (2003–2006) carried out in Galicia and the Bay of Biscay. According to the BAI, the health status of mussels was severely affected by POS and signals of recovery were evidenced in Galicia after April-04 and in Biscay Bay after April-05. The HSI (computed by an expert system) revealed high levels of environmental stress in 2003 and a recovery trend from April-04 to April-05. In July-05, the health status of mussels worsened but in October-05 and April-06 healthy condition was again recorded in almost all localities. IBR/n and IBI indicated that mussel health was severely affected in 2003 and improved from 2004 onwards. EHCC reflected a deleterious environmental condition in 2003 and a recovery trend after April-04, although a healthy ecosystem condition was not achieved in April-06 yet. Whereas BAI and HSI provide a basic indication of the ecosystem health status, star plots accompanying IBR/n and IBI provide complementary information concerning the mechanisms of biological response to environmental insult. Overall, although the integrative indices based on biomarkers show different sensitivity, resolution and informative output, all of them provide coherent information, useful to simplify the interpretation of biological effects of pollution in marine pollution monitoring. Each others’ advantages, disadvantages and applicability for ecosystem health assessment are discussed.

## Introduction

The key objectives in the assessment of marine ecosystem health are to provide information necessary to ensure maintenance of biodiversity and the integrity of marine communities, to limit human influences on living resources, to protect critical habitats and to safeguard human health. Changes in community structure and measures of chemical contamination are often used to indicate ecosystem health status but, regrettably, these responses are manifestations of damage rather than prognostic indices (Knap et al. 2002). Changes at simplest levels of biological complexity (molecular, cellular, tissue-level), which underlie effects at complex biological levels and for which causality can be established (Cajaraville et al. 1993; Knap et al. 2002), may provide early warning of ecosystem health deterioration. Biomarkers are responses at such simple levels that indicate the presence of pollutants (exposure biomarkers) or the magnitude of the biological response to pollutant exposure (effect biomarkers; McCarthy and Shugart 1990). Effect biomarkers give a general picture of the health status of the environment whereas exposure biomarkers have specificity of reaction (McCarthy and Shugart 1990). Marine pollution monitoring programs are increasingly including molecular, cell and tissue-level biomarkers, applied in combination, for the assessment of the biological effects of pollutants (Den Besten 1998; Cajaraville et al. 2000; Viarengo et al. 2000; Knap et al. 2002; Marigómez et al. 2006; Orbea et al. 2006; Zorita et al. 2007; Hylland et al. 2008; Garmendia et al. 2011a, b, c). Thus, biomarkers have provided useful mechanistic information to scientists, albeit the full potential of using biomarkers in biological monitoring programs has been limited by the scarcity of integrated statistical analysis (Beliaeff and Burgeot 2002). During the recent last years, however, biomarkers have been integrated in ecosystem health indices for simplicity purposes. The use of these indices provides comprehensive information about the biological effects of pollution in marine organisms and may therefore serve as useful tools for environmental managers (Broeg and Lehtonen 2006).

The bioeffects assessment index (BAI; Broeg et al. 2005), a modification of the “Health Assessment Index” (HAI; Adams et al. 1993), was designed for the assessment of multifactorial contamination in coastal areas using fishes as sentinels (Broeg et al. 2005). BAI is defined as a “general health” index because it comprises biomarkers of non-specific toxic effects and responds to a variety of different contaminants (Broeg et al. 2005). BAI was first applied for the long-term study of the biological effects of pollution in the German Bight using flounders (*Platichthys flesus*) as sentinels, and included deleterious effects at different levels of biological complexity, say: changes in EROD activity, LMS, NL and macrophage aggregates in liver, as well as diversity of parasitic fauna (Broeg et al. 2005). BAI was also satisfactorily applied in the Baltic Sea (Broeg and Lehtonen 2006), as a part of the EU-BEEP project, where biomarkers had been recorded in sentinel *P. flesus*, eelpouts (*Zoarces viviparous*) and blue mussels (*Mytilus edulis*). In mussels, the selected biomarkers for the calculation of BAI were LMS, NL and MN in digestive gland (Broeg and Lehtonen 2006).

The Health Status Index (HSI) is computed by an expert system (ES) designed and developed within the BEEP framework to evaluate and integrate (effect and exposure) responses of biomarkers (recorded at different levels of biological organization in mussels) to natural and contaminant-induced stress (Viarengo et al. 2000; Dagnino et al. 2007). The expert system was first applied using 11 biomarkers measured in caged mussels deployed along a pollution gradient near the Genoa Harbour (Dagnino et al. 2007). Later on, it was satisfactorily applied in several field and laboratory studies. In the field, HSI was computed to integrate seven biomarkers recorded in mussels caged along a copper pollution gradient in the Visnes fjord (Norway) (Dondero et al. 2006). The expert system was also successfully applied to a set of 8 biomarkers data from a biomonitoring study carried out about 20 years ago in the pollution gradient along the Langesundfjord (Norway) (data from GESAMP workshop (Oslo 1986); Dagnino et al. 2007). Under laboratory conditions, the expert system was employed to integrate the responses elicited in 6 biomarkers recorded in mussels exposed to crude oil, alkylated phenols and PAHs for 21 days in the RF Rogaland Research Institute (Stavanger, Norway) (Dagnino et al. 2007). In all cases, HSI computed by the expert system provided a clear indication of the stress syndrome in mussels, although the batteries of biomarkers employed differed in the type and number of biomarkers.

The Integrated Biological Response (IBR; Beliaeff and Burgeot 2002) index is based on biochemical biomarkers, including GST, AChE, CAT and ADDU. It was first applied in sentinel *P. flesus* and *M. edulis* from different areas of the Baltic Sea (Beliaeff and Burgeot 2002). IBR index was also successfully applied using four biochemical biomarkers (GST, AChE, CAT and MAD) in Canes Bay, North-Western Mediterranean Sea (Damiens et al. 2007). Bocquené et al. (2004) used IBR to combine 4 biomarkers (GST, AChE, CAT, MDA) to assess the impact of the Erika oil spill on *M. edulis* collected along the coast of Brittany (France), and demonstrated that mussels were affected for 1 year after the spill. Broeg and Lehtonen (2006), using flounders, eelpouts and blue mussels as sentinels for a pollution monitoring program in the Baltic sea, succeeded to include histochemical biomarkers (LMS, NL and MN) together with exposure biomarkers for IBR index calculation.

The Ecological Health Condition Chart (EHCC) was designed to integrate biomarker and chemical data obtained during a 2 year (1993–1994) multispecies ecotoxicological monitoring performed in the Urdaibai Reserve of the Biosphere under a contract with the Environment Department of the Basque Government (RBU-Rep 1994). Original data were protected due to contract restrictions but elaborate results were published in the form of a PhD Thesis (Díez 1996). The EHCC has been presently adapted to sentinel mussels by combining eight biomarkers. The approach consists of a graphic representation of the degree of environmental damage in a matrix chart. The color of each point depends on a graded scale (from green to red) established according to value ranges fixed considering the reference and critical values existing for each biomarker. The matrix background color is determined according to a weighted valuation of the combination of the numbers of individual biomarkers “beeping” and how much “beeps” each one (RBU-Rep 1994).

The Integrative Biomarker Index (IBI) is a new index recently developed in order to integrate biomarker data recorded within the framework of the Mussel Watch monitoring program carried out after the Prestige oil spill (POS) in Galicia and the Bay of Biscay (Marigómez et al. 2006; Orbea et al. 2006; Ortiz-Zarragoitia et al. 2011; Garmendia et al. 2011a, b, c). IBI was based on the calculation of five specific indices of deleterious effects at different levels of biological complexity: (a) Molecular/Metabolic Response Index (MRI), presently measured in terms of AOX inhibition, (AOX-effect; Garmendia et al. 2011c); (b) (sub)Cellular Response Index (CRI), measured in terms of LRI (Izagirre and Marigómez 2009, Garmendia et al. 2011a); (c) Tissue Response Index (TRI), measured in terms of Vv_BAS_ (Garmendia et al. 2011b); (d) Systemic Response Index (SRI), in terms of cumulative intensity of inflammatory responses (Garmendia et al. 2011b, c); and (e) Disease Response Index (DRI)) in terms of cumulative intensity of parasitization (Garmendia et al. 2011b, c). In order to calculate the five responses included in IBI existing reference and critical values are taken into consideration (Marigómez et al. 2006; Garmendia et al. 2010).

Thus, the present contribution is aimed at comparing different indices for biomarker integration in order to (a) determine each other’s advantages and disadvantages, as well as the convenience, reliability and environmental significance of the integrative biomarker approach; and (b) establish solid criteria for their selection depending on the user’s circumstances and capabilities.

For these purposes, the five aforementioned approaches were applied to provide an uncomplicated integrative view of the degree and duration of the POS effects assessed through biomarkers in sentinel mussels, *Mytilus galloprovincialis* collected in 22 localities along the North coast of the Iberian Peninsula over 3 years (April 2003–April 2006; Marigómez et al. 2006; Orbea et al. 2006; Cajaraville et al. 2006; Ortiz-Zarragoitia et al. 2011; Garmendia et al. 2011a, b, c). The Prestige tanker carrying 77,000 tonnes of heavy fuel–oil sunk in NW Iberian coast in November 2002. Although the Galician coast was the most impacted, the fuel–oil also affected >1,000 km coastline along the Bay of Biscay over 1 year. The profile of the long-term POS biological impact was characterized on the basis of biomarkers and tissue-level polycyclic aromatic hydrocarbons (PAHs) (Garmendia et al. 2011c). PAH (mainly naphthalene) bioaccumulation and concomitant biological effects in sentinel mussels were evident for 2 years. Sublethal effects in mussels in absence of bioaccumulation extended one more year. Putative secondary effects on mussel health status seemed to persist in April 2006, when the POS direct impact was seemingly terminated. These conclusions were based on diverse and complex data that have been presently integrated into different marine ecosystem health indices in order to provide science-based but user-friendly information for environmental managers and decision makers.

## Materials and methods

### Source data

**Fig. 1:**
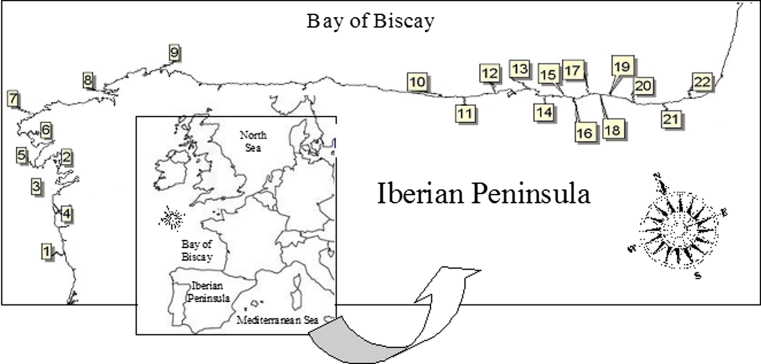
Map of the North Iberian Peninsula, showing localities where mussels, *M. galloprovincialis,* were collected. Galician Coast: 1—São Bartolomeu do Mar (41°34′36″ North; 8°48′2″ West); 2—Ons (42°22′45″ North; 8°55′42″ West); 3—Cíes (42°12′51″ North; 8°54′17″ West); 4—Oia (42°0′15″ North; 8°52′48″ West); 5—Aguiño (42°31′13″ North; 9°0′36″ West); 6—Caldebarcos (42°50′48″ North; 9°7′52″ West); 7—Camelle (43°11′38″ North; 9°5′48″ West); 8—Segaño (43°27′21″ North; 8°18′34″ West); 9—Estaca de Bares (43°45′14″ North; 7°43′24″ West). Cantabrian Coast: 10—Llanes (43°26′0″ North; 4°48′21″ West); 11—San Vicente (43°23′33″ North; 4°23′9″ West); 12—Suances (43°26′21″ North; 4°2′33″ West); 13—Pedreña (43°26′59″ North; 3°45′6″ West); 14—Laredo (43°25′0″ North; 3°24′50″ West). Basque Coast: 15—Muskiz (43°21′32″ North; 3°6′40″ West); 16—Arrigunaga (43°21′17″ North; 3°1′11″ West); 17—Gorliz (43°25′7″ North; 2°56′51″ West); 18—Bakio (43°25′57″ North; 2°48′34″ West); 19—Mundaka (43°24′16″ North; 2°41′43″ West); 20—Mutriku (43°18′11″ North; 2°21′19″ West); 21—Orio (43°17′29″ North; 2°7′30″ West); 22—Hondarribia (43°22′40″ North; 1°47′24″ West)

Previously published data obtained during a Mussel Watch monitoring carried out after POS (April 2003–April 2006) were used to construct integrative biomarker indices (Marigómez et al. 2006; Orbea et al. 2006; Cajaraville et al. 2006; Ortiz-Zarragoitia et al. 2011; Garmendia et al. 2011a, b, c). Briefly, mussels, *Mytilus galloprovincialis*, were collected along the coast of Galicia and Biscay Bay in 17 localities in April, July and September 2003 and extended to 22 localities in April, July and October (2004–2005) and April 2006 (Fig. 1). In each locality, mussels (3.5–4.5 cm shell length) were collected and pre-processed immediately after sampling, as detailed in previous reports, and further on selected biomarkers, digestive gland histopathology and gamete development were determined for each sample as summarized below.

### Biochemical biomarkers

As detailed in previous reports (Marigómez et al. 2006; Orbea et al. 2006) from which data have been obtained, the digestive gland of 10 mussels was dissected out in the field and immediately frozen in liquid nitrogen for biochemical analyses. AOX was determined spectrophotometrically. Since changes in AOX in response to POS followed a bell-shaped profile, with induction at low and inhibition at high exposure levels, two components can be distinguished: exposure (AOX_exp_) and effect (AOX_eff_) components (Garmendia et al. 2011c): AOX_exp_ = e^AOXi−AOXo^, and AOX_eff_ = e^(AOXo−AOXi)/(AOXi+1)^; where “AOX_i_” is the AOX measured and “AOX_o_” is the reference value at each season according to the available literature (Cancio et al. 1999; Garmendia et al. 2010).

### Cytochemical biomarkers

As detailed in the preceding paper (Garmendia et al. 2011a) from which data have been obtained for the present study, the digestive gland of five mussels was dissected out in the field immediately after sampling and processed to determine lysosomal responses by image analysis on cryotome sections. LP was calculated by subjective grading after the histochemical demonstration of N-acetyl-ß-hexosaminidase. Vv_L_ was determined by image analysis after the histochemical demonstration of ß-glucuronidase activity. The LRI (Izagirre and Marigómez 2009) was calculated on the basis of the LP and Vv_L_ (Garmendia et al. 2011a): LRI = √(A^2^ + B^2^); where A = [−log_2_(LP_o_/LP_i_)]; and B = [−log_2.5_(Vv_o_/Vv_i_)] (LP_o_ and LP_i_ are the reference and measured LP values, respectively; Vv_o_ and Vv_i_ are the reference and measured Vv values, respectively).

### Histological procedure and tissue-level biomarkers

As detailed in the preceding paper (Garmendia et al. 2011b) from which data have been obtained for the present study, 10 mussels were fixed *in toto* in the field in 4 % formaldehyde in 0.1 M phosphate buffer for at least 1 week and further on the digestive gland was paraffin embedded, cut on rotary microtome and stained with haematoxylin-eosin. Vv_BAS_ (μm^3^/μm^3^), MET (μm), MLR (μm) and the ratio MLR/MET (μm/μm) were calculated after quantitative microscopy (Garmendia et al. 2011b).

The Epithelial Response Index (ERI) was calculated on the basis of the Vv_BAS_ values, according to the following formulae: ERI = e^(VvBAS−i−VvBAS−o/VvBAS−o)^; where “Vv_BAS−i_” is the Vv_BAS_ measured and “Vv_BAS−o_” is the reference value, according to the available literature (Díez 1996; Marigómez et al. 2006; Garmendia et al. 2010, 2011b). Theoretically, ERI values go up to ∞ (high effect) with ERI ≤ 1 for the reference condition. However, since hitherto the highest Vv_BAS_ found are always below 0.4 μm^3^/μm^3^, practically, ERI will be always below 15.

### Digestive gland tissue histopathology

The data on the prevalence and intensity of individual inflammatory responses or parasitosis were obtained in a preceding study (Garmendia et al. 2011b), in which parasites and histopathological alterations were scored using either quantitative or semi-quantitative scales. Intensity values of these inflammatory responses and parasitic infestations were used to estimate their corresponding cumulative intensity (CI_IR_ and CI_PI_, respectively), which may provide epizootiological indication of health impairment in mussel populations (i.e. enhanced activity of the systemic immune response or augmented susceptibility to disease; Garmendia et al. 2011b): CI_IR_ = SP_IR_/NH_IR_ and CI_PI_ = SP_PI_/NH_PI_; where NH is the number of specimens presenting inflammatory responses (NH_IR_) or hosting parasites (NH_PI_), and SP is the score corresponding to each inflammatory response (SP_IR_) and parasitic infestation (SP_PI_) recorded.

In order to calculate parameters suitable to be included in ecosystem health indices, the Inflammatory Response Index (IRI) and the Parasitic Infestation Index (PII) were computed considering the recorded CI values against its putative critical values. Due to the lack of previous baseline data for CI_IR_ and CI_PI_ their critical values were arbitrarily determined. Inflammatory responses were weighted according to their severity before their integration in the CI_IR_: ×1 factor was applied to the intensity of hemocytic infiltration and brown cell aggregates, whereas a 5× factor was applied to the intensity of granulocytomas. “2” was arbitrarily established as the critical value for CI_IR_. In order to compute CI_PI_, direct individual intensities were used except for scores of *Nematopsis* that were log_10_ transformed to avoid bias of the data. As a preliminary approach, due to the lack of sufficient background data, the median of all the CI_PI_ values obtained in this study was selected as the critical value. IRI and PII were calculated according to the following formulae: IRI = CI_IR−i_/CI_IR−o_, and PII = log_2_((CI_PI−i_/CIPI − o) + 1); where CI_IR−i_ and CI_PI−i_ are the measured CI_IR_ and CI_PI_ values, and CI_IR−o_ and CI_PI−o_ are the critical CI_IR_ and CI_PI_ values, respectively. Before more substantial data are available, CI_IR−o_ has been arbitrarily fixed as “2″ and CI_PI−o_ as the median value of the recorded CI_PI−o_ (CI_PI−o_ = 1.4). IRI and PII values go up to ∞ (high effect) with IRI ≤ 1 and PII ≤ 1 for the reference condition.

### Ecosystem health indices

#### Bioeffects Assessment Index (BAI)

BAI integrates biomarker data from different biological organization level (molecular, subcellular, cellular, individual, community) by substituting each individually measured value with an arbitrary numerical value that reflects the progression of the toxically induced alterations: 10 = stage 1; 20 = stage 2; 30 = stage 3; 40 = stage 4. The BAI value for each sample is the mean value of all the numerical values assigned to individual alterations (Broeg et al. 2005). “25” has been arbitrarily determined as the critical BAI value, whereas values above 30 are indicative for an advanced state of environmental deterioration (Broeg et al. 2005). Broeg et al. (2005) fixed these values for fishes; however, although Broeg and Lehtonen (2006) also applied them successfully for mussels, they concluded that the critical values needed to be adapted to this species. Presently, in view of our data distribution, we have modified these critical values, with “20” as the critical BAI value: 10–15 = “good environmental condition”; 15–20 = ”tolerable environmental condition”; 20–30 = “delicate environmental condition”; and 30–40 = “bad environmental condition”. Following recommendations by Broeg et al. (2005), biomarkers at different level of biological complexity were used: AOX_eff_ at the molecular level; LP at the subcellular level; Vv_L_ at cellular level; Vv_BAS_ at tissue-level; CI_IR_ at individual level; CI_PI_ at the population level. The numerical values assigned to each biomarker are shown in Table 1. LP values were used as guide parameters. Due to the accidental loss of frozen material in April-03, LP values recorded in July-03 were used to complete the data matrix, aware that this might cause some weakness in the reliability of the results obtained.

**Table 1:** Stages of toxically induced alterations of the biomarkers (related to their corresponding references values): AOX_eff_ (Cancio et al. 1999; Garmendia et al. 2011c); LP (Viarengo et al. 2000; Marigómez et al. 2006; Izagirre et al. 2008; Garmendia et al. 2010, 2011a); Vv_L_ (Marigómez et al. 1996, 2006; Izagirre et al. 2008; Garmendia et al. 2010, 2011a); Vv_BAS_ (Méndez 1993; Marigómez et al. 2006; Garmendia et al. 2010, 2011b); and CI_IR_ and CI_PI_ (Garmendia et al. 2011c) and their corresponding BAI values

Parameter	Stage 1	Stage 2	Stage 3	Stage 4
AOX_eff_	0 to 1	>1 to 1.5	>1.5 to 2	>2
Numerical BAI	10	20	30	40
LP (min)	>20	>10 to 20	>5 to 10	5 to 0
Numerical BAI	10	20	30	40
Vv_L_	<0.002 μm^3^/μm^3^ (fall, summer) <0.0005 μm^3^/μm^3^ (spring)	0.002 to <0.003 μm^3^/μm^3^ (fall, summer) 0.0005 to <0.00125 μm^3^/μm^3^ (spring)	0.003 to <0.004 μm^3^/μm^3^ (fall, summer) 0.00125 to <0.002 μm^3^/μm^3^ (spring)	≥0.004 μm^3^/μm^3^ (fall, summer) ≥0.002 μm^3^/μm^3^ (spring)
Numerical BAI	10	20	30	40
Vv_BAS_	<0.1 μm^3^/μm^3^	0.1 to <0.15 μm^3^/μm^3^	0.15 to <0.2 μm^3^/μm^3^	≥0.2 μm^3^/μm^3^
Numerical BAI	10	20	30	40
CI_IR_	<0.7	0.7 to <1–4	1.4 to <2.8	≥2.8
Numerical BAI	10	20	30	40
CI_PI_	0	<1	1 to <3	≥ 3
Numerical BAI	10	20	30	40

#### Health Status Index (HSI) computed by an expert system

Biomarker data were analyzed by the Expert System 6.0 software developed by Dagnino et al. (2007). This expert system takes into consideration the possible interactions among different biological responses under stress conditions, for which biomarkers at different levels of biological complexity (from molecular to individual) are required (Dagnino et al. 2007). Once the behavioral trend (increasing, decreasing, bell-shaped) and type (general stress, exposure to metals or organic xenobiotics, genotoxicity) of the biomarkers are brought into the expert system, data are statistically analyzed by the Mann–Whitney *U* test (*p* < 0.05) automatically. The battery of biomarkers includes LP as guide although another or additional guide parameters can be selected as well. A reference locality or experimental control is necessary. Then, the expert system assigns an alteration level (0–3) to all the biomarkers and computes HSI, which discriminates five levels of health status (Dagnino et al. 2007), say: A = ”healthy”; B = ”low stress”; C = ”medium stress”; D = ”high stress”; E = ”pathological stress”.

Presently, a battery of six biomarkers (AOX_exp_, AOX_eff_, LP, Vv_L_, Vv_BAS_, CI_IR_) was brought into the expert system to calculate HSI. LP values were defined as guide parameter (due to the accidental loss of frozen material in April-03, LP values recorded in July-03 were used to complete the data matrix). Since no reference locality remained after POS, data recorded in Mundaka (a reference locality before POS; Díez 1996) in April-06 (long after the starting of the recovery; Cajaraville et al. 2006) were used as reference values. The characteristics of selected biomarkers are shown in Table 2.

**Table 2:** Characteristics of the biomarkers introduced in the expert system to compute HSI (Dagnino et al. 2007)

Biomarker	Level of biological organisation	Biological significance	Stress response profile
AOX_exp_	Cell-level	Increasing	Exposure to aromatic xenobiotics
AOX_eff_	Cell-level	Increasing	General stress
LP	Cell-level	Decreasing	General stress
Vv_L_	Cell-level	Increasing	General stress
Vv_BAS_	Tissue-level	Increasing	General stress
CI_IR_	Tissue-level	Increasing	General stress

#### Integrated Biological Response (IBR)

IBR index is based on the integration of biochemical (GST, AChE, CAT, MAD), genotoxicity (ADDU) and histochemical (LP, NL, MN) biomarkers (Beliaeff and Burgeot 2002). The calculation method is based on relative differences between the biomarkers in each given data set. Thus, the IBR index is computed by summing-up triangular star plot areas (multivariate graphic method) for each two neighboring biomarkers in a given data set, according to the following procedure: (1) calculation of the mean and standard deviation for each sample; (2) standardization of data for each sample: x_i_′ = (x_i_−x)/s; where, x_i_′ = standardized value of the biomarker; x_i_ = mean value of a biomarker from each sample; x = general mean value of x_i_ calculated from all compared samples (data set); s = standard deviation of x_i_ calculated from all samples; (3) addition of the standardized value obtained for each sample to the absolute standardized value of the minimum value in the data set: y_i_ = x_i_′ + |x_min_′|; (4) calculation of the Star Plot triangular areas as A_i_ = (y_i_ × y_i+1_×sin*α*)/2, where “y_i_” and “y_i+1_” are the standardized values of each biomarker and its next biomarker in the star plot, respectively, and “*α*” is the angle (in radians) formed by each two consecutive axis where the biomarkers are represented in the Start Plot (*α* = 2π*/*n; where “n” is the number of biomarkers); and (5) calculation of the IBR index which is the summing-up of all the Star Plot triangular areas (IBR = ∑A_i_) (Beliaeff and Burgeot 2002). Since the IBR value is directly dependent on the number of biomarkers in the data set, the obtained IBR value must be divided by the number of biomarkers used (IBR/n; Broeg and Lehtonen 2006).

Presently, five biomarkers (AOX_eff_, LP, Vv_BAS_, CI_IR_, and CI_PI_) were integrated in the IBR index calculated as IBR/n, according to Broeg and Lehtonen (2006). LP values recorded in July-03 were used to replace missing LP values in April-03.

#### Ecosystem health condition chart (EHCC)

EHCC is a graphic representation of the degree of environmental damage in a matrix chart (RBU-Rep 1994). Presently, EHCC was produced on the basis of a battery of eight selected exposure and effect biomarkers (AOX_exp_, AOX_eff_, LP, Vv_L_, Vv_BAS_, MLR/MET, CI_IR_, CI_PI_) to characterize the ecosystem health condition of each sample (locality and sampling time) after POS. A color grading scale was assigned to each biomarker depending on the degree of environmental deterioration they indicated (Table 3): green (stage 1) = ”good ecosystem health condition”; yellow (stage 2) = ”tolerable ecosystem health condition”; orange (stage 3) = ”delicate ecosystem health condition”; and red (stage 4) = ”bad ecosystem health condition”. Grading was established according to the existing data on reference and critical values, based on the RBU-Rep (1994) and Marigómez et al. (2004, 2006), as well as in the literature available for specific biomarkers, say: AOX_exp_ and AOX_eff_ (Cancio et al. 1999), LP (Viarengo et al. 2000; Izagirre et al. 2008), Vv_L_ (Marigómez et al. 1996; Izagirre et al. 2008), Vv_BAS_ and MLR/MET (Cajaraville et al. 1991; Méndez 1993; Díez 1996), CI_IR_ and CI_PI_ (present work). Finally, the ecosystem health condition for each sample was determined by integrating the signals provided by individual biomarkers, according to the criteria detailed in Table 4. Thus, the background color of the matrix for each sample (set of color spots) results from the weighted valuation of the combination of which and how many individual biomarkers are giving a warning sign and the magnitude of each sign (RBU-Rep 1994).

**Table 3:** Color graduation stage for each biomarker according to the progression of environmental deterioration

Parameter	Green (stage 1)	Yellow (stage 2)	Orange (stage 3)	Red (stage 4)
AOX_exp_	<1	1 to < 1.5	1.5 to <2	≥2
AOX_eff_	<1	1 to < 1.5	1.5 to <2	≥2
LP	>20 min	>10–20 min	>5–10 min	≤5 min
Vv_L_	<0.002 μm^3^/μm^3^ (fall, summer) <0.0005 μm^3^/μm^3^ (spring)	0.002 to <0.003 μm^3^/μm^3^ (fall, summer) 0.0005 to <0.00125 μm^3^/μm^3^ (spring)	0.003 to <0.004 μm^3^/μm^3^ (fall, summer) 0.00125 to <0.002 μm^3^/μm^3^ (spring)	≥0.004 μm^3^/μm^3^ (fall, summer) ≥0.002 μm^3^/μm^3^ (spring)
Vv_BAS_	<0.1 μm^3^/μm^3^	0.1 to <0.15 μm^3^/μm^3^	0.15 to <0.2 μm^3^/μm^3^	≥0.2 μm^3^/μm^3^
MLR/MET	<1.2 μm/μm (fall, summer) <0.6 μm/μm (spring)	1.2 to <1.4 μm/μm (fall, summer) 0.6 to <1 μm/μm (spring)	1.4 to <1.6 μm/μm (fall, summer) 1 to <1.4 μm/μm (spring)	≥1.6 μm/μm (fall, summer) ≥1.4 μm/μm (spring)
CI_IR_	<0.3	0.3 to <0.7	0.7 to <1.3	≥1.3
CI_PI_	< 0.7	0.7 to <1.4	1.4 to <2.8	≥2.8

Scales were established according to references values (AOX_eff_ and AOX_exp_ (Cancio et al. 1999; Garmendia et al. 2010, 2011c); LP (Viarengo et al. 2000; Marigómez et al. 2006; Izagirre et al. 2008; Garmendia et al. 2010, 2011a); Vv_L_ (Marigómez et al. 1996, 2006; Izagirre et al. 2008; Garmendia et al. 2010, 2011a); MLR/MET and Vv_BAS_ (Méndez 1993; Garmendia et al. 2010, 2011b); and CI_IR_ and CI_PI_ (Garmendia et al. 2011c). *Green* good environmental condition, *yellow* tolerable environmental condition, *orange* delicate environmental condition, *red* bad environmental condition (RBU-Rep 1994)

**Table 4:** Criteria employed to determine the environmental condition for each sample on the basis of the additive integration of its colour graded spots (RBU-Rep 1994)

Number of warning “lights” over 8	Ecosystem health condition
4 Yellow	Good (green)
2 Orange	
3 Yellow + 1 orange	
1 Red	
5 Yellow	Tolerable (yellow)
3 Orange	
4 Yellow + 1 orange	
3 Yellow + 2 orange	
2 Yellow + 2 orange	
3 Yellow + 1 red	
8 Yellow	Delicate (orange)
4 Orange	
3 Orange + 2 yellow	
2 Orange + 4 yellow	
1 Orange + 6 yellow	
2 Orange + 1 red	
2 Red	
Red (LP)	Bad (red)
6 Orange	
3 Red	
Red (LP) + 4 yellow	
Red (LP) + 2 orange	
Red (LP) + 1 red	
2 Red + 3 orange	
2 Red + 6 yellow	

#### Integrative Biomarker Index (IBI)

IBI integrates biomarkers and provide a comprehensive indication of the degree and duration of environmental damage. IBI is based on the calculation of indices of deleterious effects at five different levels of biological complexity: Molecular/Metabolic Response Index (MRI), (sub)Cellular Response Index (CRI), Tissue Response Index (TRI), Systemic Response Index (SRI), and Disease Response Index (DRI). Thus, IBI is computed by summing-up triangular star plot areas for each two neighboring response indices in a given data set, according to the following procedure: (1) standardization of data for each sample: in order to calculate responses indices, existing reference and critical values are taken into consideration (their calculation must be formulated in a way that “0” corresponds to reference and “1” to critical values); (2) calculation of the Star Plot triangular areas as A_i_ = (y_i_ × y_i+1_×sin*α*)/2, as above detailed for IBR; and (3) calculation of IBI, which is the summing-up of all the Star Plot triangular areas (IBI = ∑A_i_).

Presently, MRI was measured in terms of AOX_eff_, CRI in terms of LRI, TRI in terms of ERI, SRI in terms of IRI and DRI in terms of PII; and the IBI was calculated as above described. LRI values recorded in July-03 were used to replace missing LRI values in April-03.

## Results

### Bioeffects Assessment Index (BAI)

**Fig. 2:**
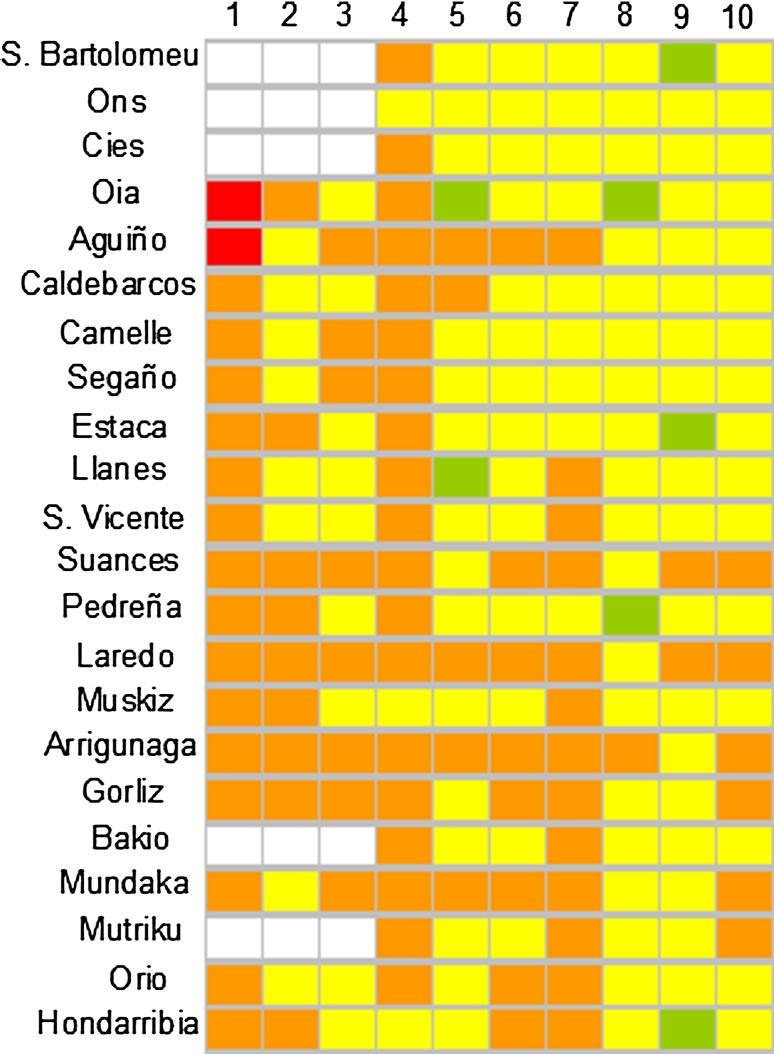
Diagrammatic representation of the BAI categories (*White* sample missing/lost, *Green* good environmental condition, *Yellow* tolerable environmental condition, *Orange* delicate environmental condition, *Red* bad environmental condition) determined using mussels *M. galloprovincialis* as sentinels to monitor ecosystem health after POS in Galicia and the Bay of Biscay. *1* April-03, *2* July-03, *3* Sept-03, *4* April-04, *5* July-04, *6* = Oct-04, *7* April-05, *8* July-05, *9* Oct-05, *10* April-06

High BAI values were recorded after POS in all the localities in April-03, with highest values (“bad environmental condition”) in Aguiño and Caldebarcos and “delicate environmental condition” values in every other locality (Fig. 2). BAI values decreased in July-03 in Galicia (except in Estaca) and in Llanes, San Vicente, Mundaka and Orio in Biscay Bay. In September-03 “delicate environmental condition” was detected in Aguiño, Camelle and Segaño in Galicia and in Suances, Laredo, Arrigunaga, Gorliz and Mundaka in Biscay Bay; whereas “tolerable environmental condition” was assigned to the remainder localities (Fig. 2). In April-04, BAI values increased and “delicate environmental condition” was recorded in all the studied localities except in Ons, Muskiz and Hondarribia (Fig. 2). Later on, since July-04 to April-06 a recovery trend was envisaged first in Galicia (“delicate environmental condition” only in Aguiño until April-05 and in Caldebarcos until July-04) and then in Biscay Bay (“delicate environmental condition” in all localities but Pedreña until April-05), with most localities presenting BAI values corresponding to “good” or “tolerable environmental condition”. Exceptionally, Arrigunaga in July-05 and Suances and Laredo in October-05 were subjected to “delicate environmental condition”. In April-06, however, BAI indicated “delicate environmental condition” in some localities in Biscay Bay (Suances, Laredo, Gorliz, Mundaka and Mutriku) (Fig. 2).

### Health Status Index (HSI)

**Fig. 3:**
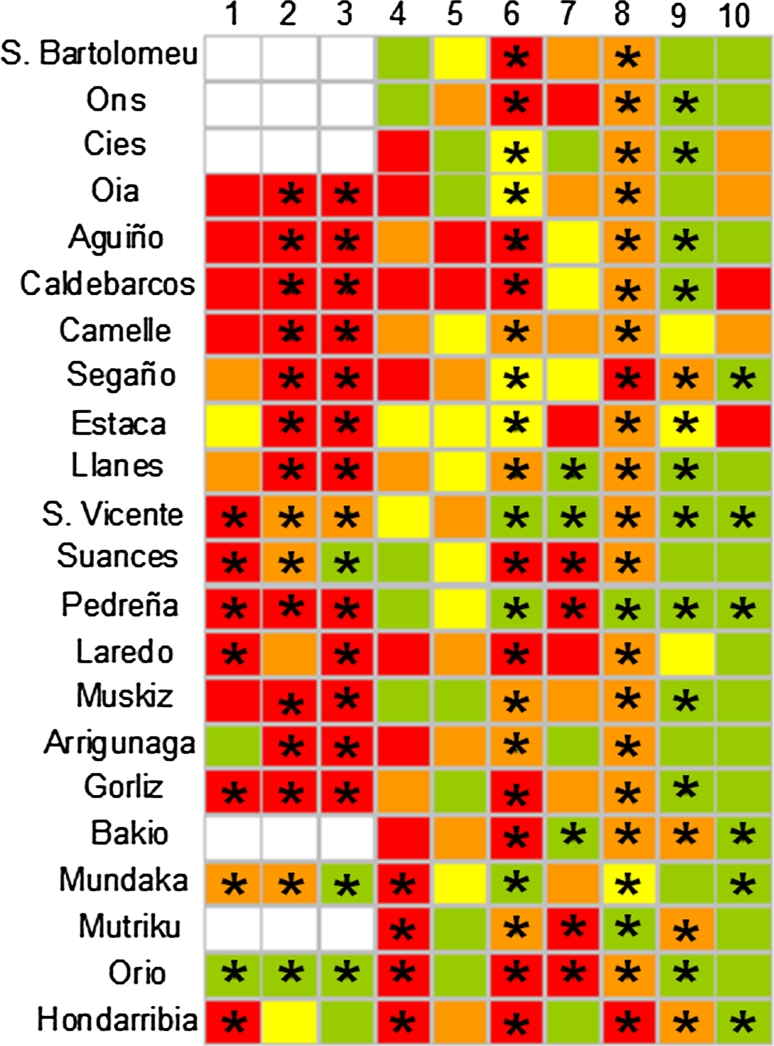
Diagrammatic representation of the Health Status Index (HSI) (*White* sample missing/lost, *Green* healthy, *Yellow* low stress, *Orange* Medium stress, *Red* high stress) determined by the expert system using mussels *M. galloprovincialis* as sentinels to monitor ecosystem health after POS in Galicia and the Bay of Biscay. Asterisks indicate exposure to organic chemical compounds, according to the exposure biomarker AOX_exp_. *1* April-03, *2* July-03, *3* Sept-03, *4* April-04, *5* July-04, *6* Oct-04, *7* April-05, *8* July-05, *9* Oct-05, *10* April-06

The Expert System did not identify any case of “pathological health status” but revealed “high environmental stress” in April-03, July-03 and September-03 in most localities (Fig. 3). After April-04, HSI values decreased and the “medium stress” was dominant in July-05 and the “healthy condition” after October-05. However, “high stress” was still detected in: (a) Cíes, Oia, Caldebarcos and Segaño in Galicia, and Laredo, Arrigunaga, Bakio, Mundaka, Mutriku, Orio and Hondarribia in Biscay Bay in April-04; (b) Aguiño and Caldebarcos in July-04; (c) São Bartolomeu do Mar, Ons, Aguiño, Caldebarcos, Suances, Laredo, Gorliz, Bakio, Orio and Hondarribia in October-04; (d) Ons, Estaca, Suances, Pedreña, Laredo Mutriku and Orio in April-05; (e) Segaño and Hondarribia in July-05; and (f) Caldebarcos and Estaca in April-06 (Fig. 3). In addition, according to AOX_exp_, the expert system revealed exposure to organic xenobiotics in Biscay Bay localities in April-03, in all localities in July-03, September-03, October-04 and July-05, in Mundaka, Mutriku, Orio and Hondarribia in April-04, and in all localities except in São Bartolomeu do Mar, Laredo, Arrigunaga and Mundaka in October-05 (asterisks in Fig. 3). Llanes, San Vicente, Suances, Pedreña, Bakio, Mutriku and Orio in April-05 and Segaño, San Vicente, Pedreña, Bakio, Mundaka and Hondarribia in April-06 were subjected to exposure to organic xenobiotics, according to AOX_exp_ (Fig. 3).

### Integrative Biological Response (IBR)

**Fig. 4:**
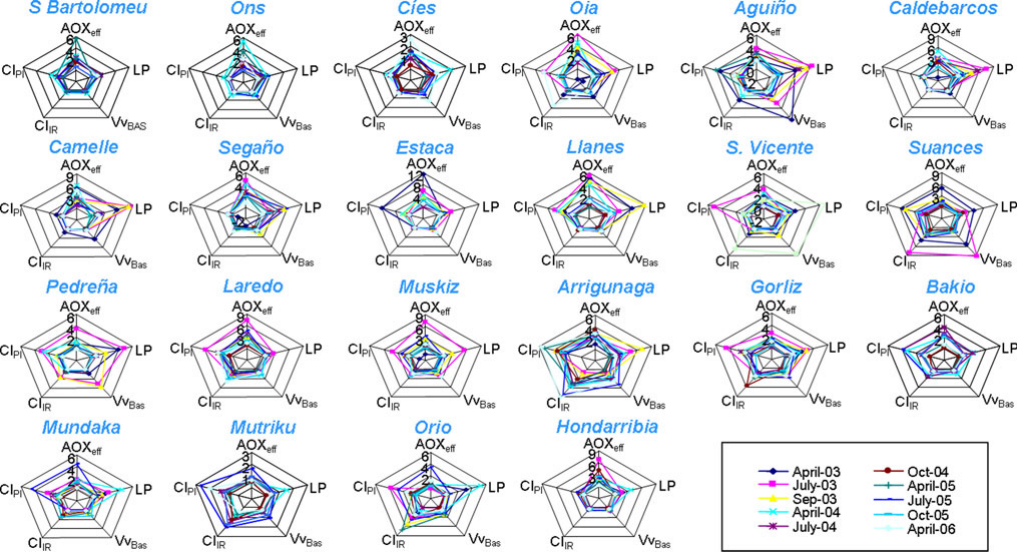
Star plots representing the five biomarkers (AOX_eff_, LP, Vv_BAS_, CI_IR_ and CI_PI_) used to compute the IBR/n index that were measured in localities studied during the biological Mussel Watch programme carried out to monitor ecosystem health after POS in Galicia and Biscay Bay (2003–2006). Each of the five axes of the star plots represents the relative degree of response of one biomarker. *Colour* *lines* represent different samplings (legend)

**Fig. 5:**
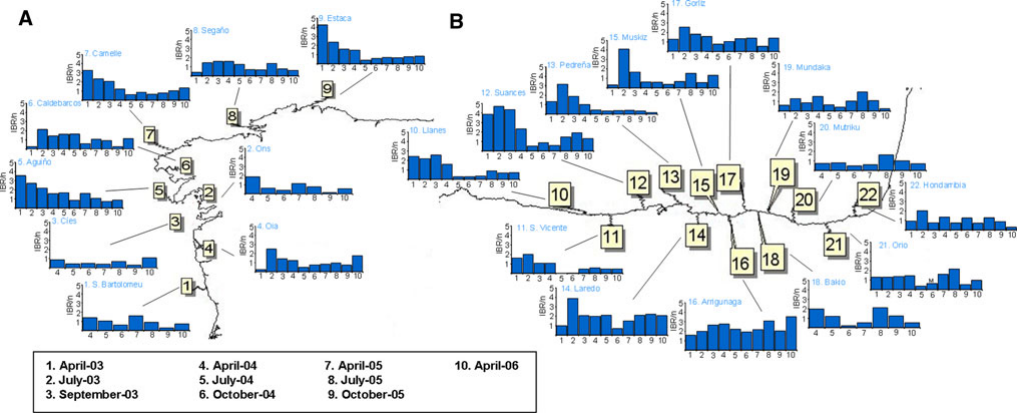
IBR/n index in mussels *M. galloprovincialis* from Galicia (**a**) and the Bay of Biscay (**b**) after POS. Five biomarkers (AOX_eff_, LP, Vv_BAS_, CI_IR_ and CI_PI_) were used to compute the IBR/n index

Five biomarkers (AOX_eff_, LP, Vv_BAS_, CI_IR_, CI_PI_) were represented in start plots (Fig. 4); in which the relative degree of response for each biomarker is represented in the corresponding axis for the different samplings. For instance (Fig. 4), the degree of LP response (less complex biological level) is high in Caldebarcos in April-September 2003 whilst it is low in Arrigunaga together with a high degree of response in most complex biological levels (CIIR and CIPI). Thus, AOX_eff_ and LP were the most sensitive biomarkers in most localities since April-03 to April-04, except in Aguiño where Vv_BAS_ was dominant in April-03, Estaca where AOX_eff_, and CI_PI_ were dominant in April-03, San Vicente, Laredo, Gorliz, Orio where CI_PI_ was dominant in July-03 and Suances where CI_IR_ and Vv_BAS_ were dominant in July-03 (Fig. 4). After April-04, standardized biomarker values remained low and balanced except in San Vicente in April-06, where CI_PI_ and Vv_BAS_ were dominant; Arrigunaga, where CI_IR_ and CI_PI_ were dominant until April-06; Gorliz in October-04 and Orio in April-05, where CI_IR_ was dominant; and Mundaka in July-05 where CI_PI_ was dominant (Fig. 4). Overall, IBR/n values were higher in 2003 and April-04 than in the remainder sampling times in almost all localities (Fig. 5). In contrast, Laredo and Arrigunaga showed moderately high-to-high values continuously all along the studied period. Eventually, IBR/n values raised transiently in July-05 in Segaño, Muskiz, Mundaka, Mutriku, Bakio and Orio (Fig. 5).

### Ecological health condition chart (EHCC)

**Fig. 6:**
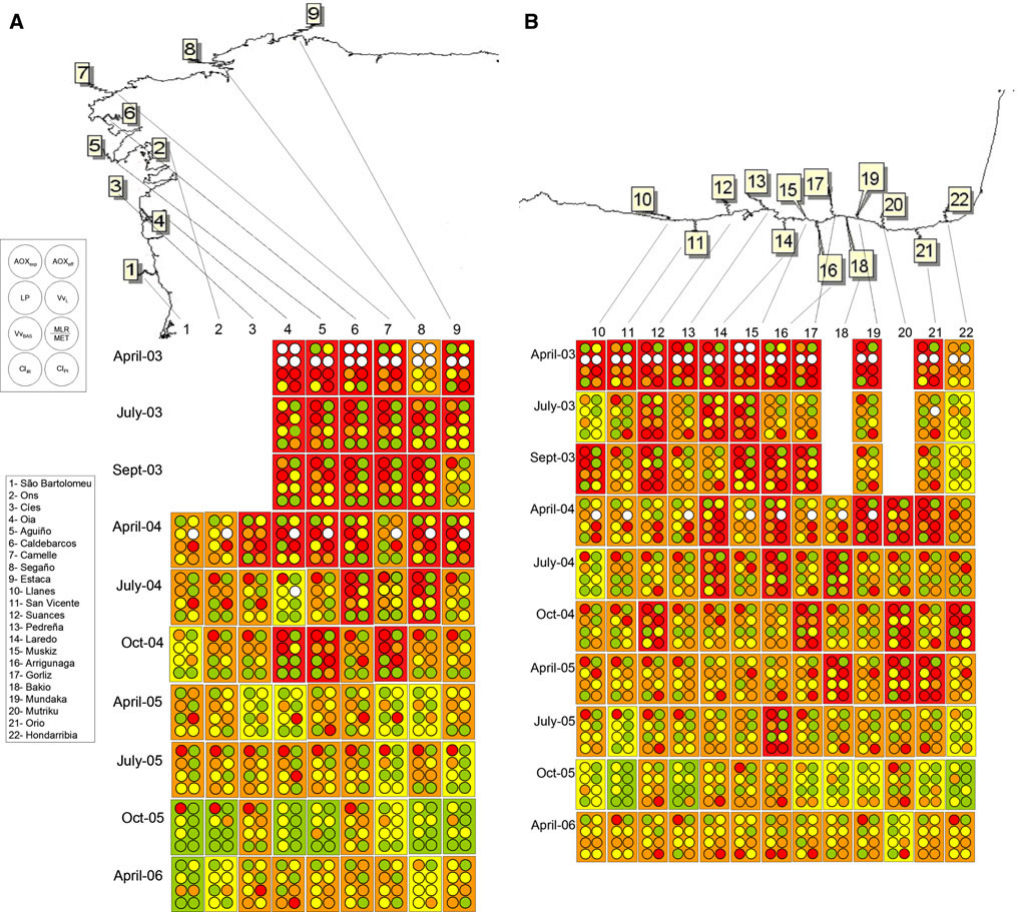
EHCCs performed using eight biomarkers (AOX_exp_, AOX_eff_, LP, Vv_L_, MLR/MET, Vv_BAS_, CI_IR_ and CI_PI_) measured in mussels *M. galloprovincialis* collected after POS in Galicia (**a**) and the Bay of Biscay (**b**). For each sample, each* colour *spot corresponds to a single biomarker, as indicated in the left top legend, and the background colour in each rectangle corresponds to the ecosystem health condition (*White* sample missing/lost, *Green* good ecosystem health condition, *Yellow* tolerable ecosystem health condition, *Orange* delicate ecosystem health condition, *Red* bad ecosystem health condition), according to grading criteria indicated in Tables 3 and 4

EHCC showed “bad ecosystem health condition” in most of the localities in 2003 and April-04 (Fig. 6). Signals of recovery started sooner in Galicia, where “bad ecosystem health condition” was only detected in Caldebarcos and Segaño in July-04 and in Oia and Camelle in October-04 (Fig. 6A). In Biscay Bay, “bad ecosystem health condition” was recorded until October-05 in almost all localities (Fig. 6B). “Delicate ecosystem health condition” was recorded in most localities after April-04. The healthiest localities were Estaca and San Vicente, which only presented a “tolerable ecosystem health condition” in October-05. Overall, although most biomarkers except AOX_exp_, MLR/MET, CI_IR_ and CI_PI_, showed signals of recovery since April-05, “delicate ecosystem health condition” was found in all localities until April-06 (Fig. 6).

### Integrative Biomarker Index (IBI)

**Fig. 7:**
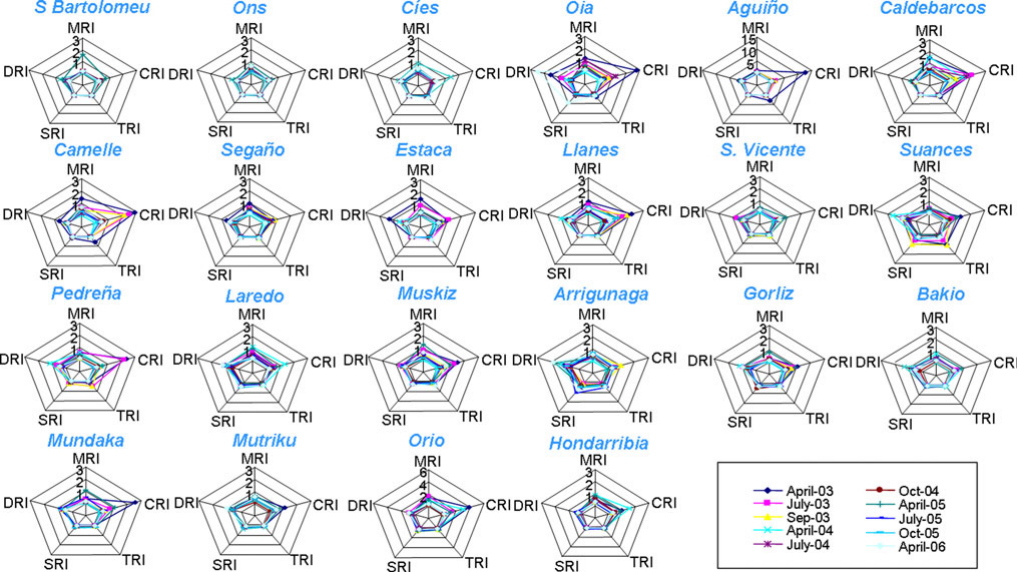
Star plots representing the responses at the five levels of biocomplexity (MRI = AOX_eff_, CRI = LRI, TIR = ERI, SRI = IRI and DRI = PII) used to compute the IBI that were estimated after measuring biomarkers (AOX_eff_, LP, Vv_L_, Vv_BAS_, CI_IR_ and CI_PI_) in mussels from the localities studied during the biological Mussel Watch programme carried out to monitor ecosystem health after POS in Galicia and the Bay of Biscay (2003–2006). Each of the five axes of the star plots represents the relative degree of response of one biomarker. *Colour* *lines* represent different samplings (legend)

**Fig. 8:**
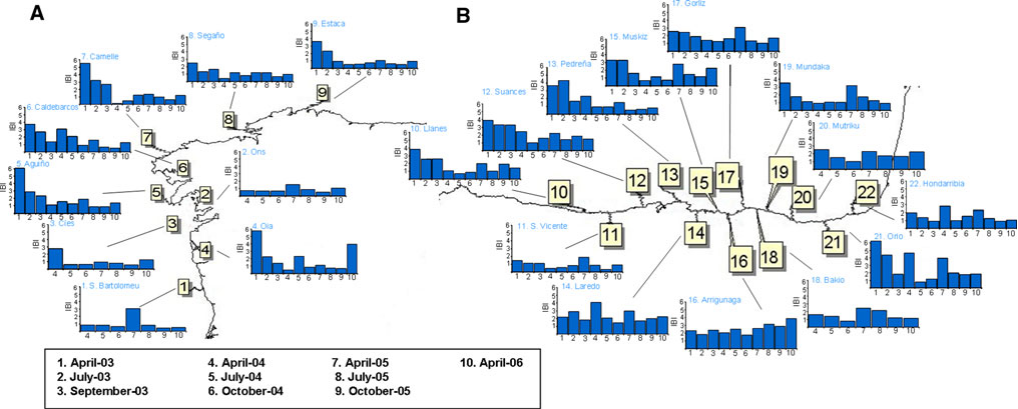
IBI in mussels *M. galloprovincialis* from Galicia (**a**) and the Bay of Biscay (**b**) after POS. Five indices of the biological response recorded in mussels at different levels of biological complexity (MRI, CRI, TRI, SRI and DRI) were used to compute the IBI

Five indices of biological response (MRI, CRI, TRI, SRI, DRI) were represented in start plots (Fig. 7). CRI was the most sensitive biological response, mainly in 2003 and in April-04 (Fig. 7). TRI was also dominant in Aguiño in April-03 and DRI in Suances in April-04 and in Arrigunaga in April-05 and April-06 (Fig. 7). Overall, IBI values were higher in 2003 and April-04 than in the remainder sampling times in almost all localities (Fig. 8). In contrast, Laredo and Arrigunaga showed moderately high-to-high values continuously all along the studied period. Occasionally, IBI values raised transiently in most of the localities in April-05 and/or July-05 (Fig. 8).

## Discussion

### Integrative assessment of POS effects

#### Bioeffects Assessment Index (BAI)

According to the BAI, ecosystem health was highly affected by POS in all the study area in April-03, and most severely in Aguiño and Caldebarcos. The ecosystem health status improved slightly in most localities in July-03 and less markedly in September-03. In April-04, “delicate environmental condition” was recorded again in most localities, which would not be attributed to seasonal variability as we included seasonal reference values, according to Broeg and Lehtonen (2006). Further on, a recovery was envisaged first in Galicia and later on in the Bay of Biscay, with most localities presenting BAI values corresponding to “good” or “tolerable environmental condition”. However, only the ecosystem health status of a few localities was classified as “good condition”.

Therefore, BAI was useful to determine different ecosystem health status in different localities at different times and, overall, revealed POS impact in 2003 and further recovery with some eventual exceptions. However, its discrimination power was limited (condition of most samples was recognized as either “tolerable” or “delicate”, with a few characterized as either “good” or “bad”) and more solid reference values and optimization of the ranges used to define the health status stages for some parameters (i.e. AOX_eff_, CI_IR_ and CI_PI_) are needed to improve it. It was previously stated that the critical BAI value used for fishes (“25”; Broeg et al. 2005) is not fully adequate for mussels (Broeg and Lehtonen 2006). More detailed information about the effects of the alterations of single biomarkers on population health is needed to characterize an adequate critical BAI value for mussels. Meanwhile, we arbitrarily decided, after a trial-and-error approach, to reduce the critical value for mussels to “20”, which most likely is still not optimal but has been presently useful. As far as we know, BAI has been only once more applied to mussels (including LP, NL and MN, as biomarkers), aimed to discriminate different ecosystem health conditions along a pollution gradient in the Baltic Sea (Broeg and Lehtonen 2006). In agreement with our present results, the BAI discrimination power was also limited in that work, and only conditions corresponding to BAI values over or below the critical value were distinguished. On the other hand, in a attempt to improve the resolution of BAI, a new index (Biomarker Response Index -BRI-; Hagger et al. 2008) was developed by adapting BAI to categories used under the European Water Framework Directive (WFD) for ecological and chemical assessment (Hagger et al. 2008). BRI was applied to compare the health of mussels from 10 British estuaries affected by the WFD, concluding that eight sites were healthier than predicted and two showed a similar health status to that of the predicted point-source pollution risk classification, which highlighted the interest of implementing BRI within WFD endpoints (Hagger et al. 2008).

#### Health Status Index (HSI)

HSI did not reveal “pathological health status” in any case, although “high environmental stress” was found in 2003 in most localities. After April-04, ecosystem health status improved resulting in a dominant “healthy condition” from October-05 onwards, although “medium” or “high stress” was occasionally evidenced in a few localities. HSI showed that the ecosystem health status varied largely among localities in 2004 but became more or less uniform for the entire study area since July-05. Besides, according to AOX_exp_, the expert system revealed exposure to organic xenobiotics in April-03 in some localities of Galicia and the Bay of Biscay and in most localities in summer/autumn after October-04. AOX_exp_ was not sensitive in Galicia in April-03 due to severe metabolic toxic damage, as revealed by the low AOX levels and high AOX_eff_ values recorded at this sampling time (Orbea et al. 2006).

Therefore, HSI was useful to determine different ecosystem health status in different localities at different times and, overall, revealed POS impact in 2003 and further recovery with some sporadic exceptions. Although the most critical stage (“pathological condition”) was not assigned to any sample, the discrimination power of HSI allowed us to recognize “healthy”, “low stress”, “medium stress” and “high stress” conditions regarding ecosystem health status after POS. Alas, no clear direct relationship between exposure (AOX_exp_) and health condition (HSI) was found. Although clear dose–response relationships and causality have been often demonstrated for individual biomarkers and single pollutants under controlled laboratory conditions and relatively short-term exposures, the lack of correspondence between AOX_exp_ and HSI is not unexpected. On the one hand, AOX_exp_ was not sensitive in Galicia in April-03 due to severe metabolic toxic damage, as above mentioned (Orbea et al. 2006), which explains the blanks in the first sampling in Fig. 3. On the other hand, AOX_exp_ was correlated positively with some biomarkers used to compute HSI (Vv_L_) but negatively with others (AOX_eff_, Vv_BAS_, and CI_IR_) (Garmendia et al. 2011c), which might result in attenuated co-variability between AOX_exp_ and HSI. Moreover, although these significant correlations were essentially explained by the remarkable alterations recorded in 2003–2004 together with highest tissue PAH levels, successive impacts of different nature were reported to occur after POS (Garmendia et al. 2011c): (a) PAH bioaccumulation and concomitant biological effects in 2003–2004; (b) persistent sublethal effects in absence of bioaccumulation (e.g. impaired health status of previously affected individuals) in 2005; and (c) secondary effects on mussel health emerging after POS impact cessation (at least until April 2006). These long-term trends would explain apparent inconsistencies between AOX_exp_ and HSI. It is also worth noting that each biomarker possesses distinct adaptive and recovery capacities and response times (Wu et al. 2005); which depend on the environmental conditions and may be modified by the presence in the field of multiple stress sources acting in combination. Consequently, causality cannot be established assuming simple dose–response relationships; for which relating HSI to exposure biomarkers such as AOX_exp_ may be unhelpful in long-term field studies. However, the ecosystem health impairment after POS is irrefutably shown by HSI, which is the main goal in monitoring the biological effects of pollutants. Alternatively, HSI (like any other index in this study) could be combined with other approaches (i.e. weight-of-evidence; Chapman, 2007), to establish causality.

This expert system was previously applied to mussels including different suites of biomarkers. In a first study, LP, NL, LPF, lysosomal/cytoplasm volume ratio -volume density according to Weibel (1979)- (eq. Vv_L_), Ca^2+^-ATPase, CAT, and MT were integrated as HSI to assess ecosystem health along a pollution gradient in the Visnes fjord in Norway (Dondero et al. 2006). “Healthy”, “low stress” and “high stress” conditions were distinguished at different sites in agreement with the existing pollution gradient. The “pathological stress” condition, however, was not assigned to any site, like in the present study. In order to recognize the “pathological stress” condition a significant response must be scored for biomarkers at the individual level (Dondero et al. 2006), which seems not to be the case nor in Visnes fjord study neither in ours. In a second study, mussels were caged in the vicinity of the Genoa harbor Oil terminal in the Ligurian sea and the biomarkers LP, NL, LPF, DNA damage, MN, CAT, GST, MT, AChE, Vv_L_, and SOS were introduced into the expert system (Dagnino et al. 2007). In this case, a “pathological stress” condition was observed after 30 days caging in a heavily polluted site. A third investigation dealt with the study of mussels sampled along the Langesund fjord in Norway, where LP, NL, LPF, GST, MT, AChE, NADPH-cyt c reductase, Vv_L_ and SFG were integrated in the HSI (Dagnino et al. 2007). Here, two sites, where human activity was highest and water exchange rate very low, were categorized as “pathologically stressed”, and a “high stress” condition was found in another site, whereas a “healthy” condition was recognized to the reference site (Dagnino et al. 2007). Finally, in mussels exposed to crude oil, alkyl phenols and PAHs under mesocosm conditions in Stavanger (Norway), the biomarkers LP, NL, LPF, MT, Vv_L_, and SOS were introduced in the expert system to compute HSI (Dagnino et al. 2007). The expert system recognized high stress levels after 21 days exposure for the three types of pollutants investigated and healthy condition for experimental controls.

In general terms, the results obtained in these four studies and their interpretation were comparable to those presently achieved. Nevertheless, we must be cautious since the apparent absence of a “pathological stress” condition after POS does not necessarily imply that such severe damage did not occur. The reference critical values of the biomarkers at the individual level presently used (CI_IR_) are not sufficiently established, and recognition of the “pathological stress” condition depends on individual level biomarkers (Dondero et al. 2006). For this reason, more deep knowledge on pollution-induced inflammatory responses and pathological lesions is needed as they can be indicative of the (individual/population) systemic/disease condition without additional samples/processing (crucial issue in pollution monitoring programs), as they are determined on the same paraffin sections used to measure tissue-level biomarkers (Vv_BAS_, MLR/MET; Marigómez et al. 2006; Orbea et al. 2006; Garmendia et al. 2011b). Alternatively, although they would demand additional samples and processing, biomarkers at the individual/population level such as SOS (Viarengo et al. 1995) might be included into the battery of biomarkers employed to compute HSI in the expert system (Dagnino et al. 2007).

#### Integrative Biomarker Response (IBR)

Five biochemical, histochemical and histological biomarkers of effect (AOX_eff_, LP, Vv_BAS_, CI_IR_, CI_PI_) were used to calculate the IBR index developed by Beliaeff and Burgeot (2002). Aware that different biomarker arrangements on the star plots produce different IBR/n values (Broeg and Lehtonen 2006) and seeking biological coherence, biomarkers were orderly represented in the five axes of start plots from the less (AOX_eff_) to the most complex (CI_PI_) biological level. Overall, highest IBR/n values were scored in 2003 and April-04, although Laredo and Arrigunaga showed moderately high-to-high IBR/n values all along the studied period and IBR/n values raised transiently in July-05 in a few localities (Segaño, Muskiz, Mundaka, Mutriku, Bakio and Orio).

Star plots revealed details about the biological responses elicited at each sampling time and locality. Effects at the simplest levels of biological complexity, such as enzyme inhibition (AOX_eff_), destabilization of the lysosomal membrane (LP) and, eventually, changes in cell type composition (Vv_BAS_), were first recorded (2003 and April-04). During this period, biomarkers at the individual/population level, such as CI_PI_ and CI_IR_, contributed eventually to IBR in a few localities in Biscay Bay (San Vicente, Suances, Laredo, Gorliz and Orio in July-03,). Interestingly, these are very touristic localities in the study area and hence they are subjected to increased anthropogenic pressure during summer time, which might enhance parasitization and associated inflammatory responses. Further on, after April-04, the responses at tissue and individual/population level gained relevance in Biscay Bay, particularly in Arrigunaga and eventually in Gorliz (October-04), Orio (April-05), Mundaka (July-05) and San Vicente (April-06), but most biomarkers remained lowered and balanced in Galicia. Exceptionally, molecular responses were dominant in S. Bartolomeu in April-05, and biomarkers at the individual/population levels were dominant in Oia in April-06. Thus, Arrigunaga might represent a chronically polluted site, S. Bartolomeu maybe some eventual episode of environmental distress of local entity, and most other cases would correspond to spring, a season where susceptibility to disease might be favored by reproductive stress under particular environmental conditions (Garmendia et al. 2010).

IBR was previously applied to fishes and mussels including different suites of biomarkers. In a first study, AChE, GST and CAT were measured in mussels collected at different sites in the Baltic Sea at different times, and AChE, GST, EROD and ADDU in flounders (*P. flexus*) collected along a pollution gradient in the Seine estuary. In both cases, star plots of the biomarkers were interpreted and the IBR index calculated in order to assess ecosystem health (Beliaeff and Burgeot, 2002). Polluted and less polluted sites were distinguished in both studies, although no definitive causal relationship was established after comparing the star plots corresponding to biomarkers and to specific pollutant levels (PCBs, PAHs). IBR index offered a useful indication of environmental stress, even though pollution was very diffuse and not attributable to one family of contaminants (Beliaeff and Burgeot 2002). Seasonal variability in IBR index was investigated in mussels (*M. galloprovincialis*; *M. edulis*) and clams (*Macoma balthica*) (Bodin et al. 2004, Leiniö and Lehtonen, 2005). IBR/n index raised in spring-early summer due to the existence of a stress syndrome related to the reproductive cycle. However, IBR/n index succeeded in identifying temporal and spatial fluctuations in ecosystem health status and their magnitude after applying different suites of biomarkers to the fishes *P. flexus* and *Z. viviparus* (LP, MN, NL, MMCs size and phosphatase activity) and to the mussel *Mytilus* spp. (LP, MN, NL, AChE and MT) collected from four localities in the Baltic Sea (Broeg and Lehtonen 2006). The IBR approach was also used in a transplant experiment in the Bay of Cannes (Mediterranean Sea), where mussels (*M. galloprovincialis*) were caged for 1 month in June (2003-2005) at several stations with different pollution levels (Damiens et al. 2007). Five biomarkers (AChE, GST, CAT, MT and thiobarbituric reactive substances) were used to construct the star plots and compute the IBR index, and the tissue concentrations of Cu, Zn, Cd, PAHs and PCBs were also measured. IBR values were up to 10 times higher in the polluted sites than in the reference site. Moreover, after comparing the star plots of IBR and pollutant concentrations, Damiens et al. (2007) found a reasonable agreement between Cu and PCB gradients and IBR variation whereas the PAH gradient did not appear related to the IBR index. Star plots also revealed that other contaminants besides Cu and PCBs contributed to high IBR values. Pytharopoulou et al. (2008) applied IBR, based on six biomarkers (MN, MT, LP, TBARS, superoxide radical production and in vitro activity of ribosomes), to *M.* *galloprovincialis* caged for 1 month in three localities of the Gulf of Patras (Mediterranean Sea) at three different seasons. IBR clearly distinguished the pollution gradient independently from the season. Star plots revealed that, especially in winter, Cr and Zn contributed to ecosystem health deterioration in some localities (high IBR values) (Pytharopoulou et al. 2008). Most recently, in mussels exposed to produced water under laboratory conditions, IBR/n demonstrated sensitivity to the complex mixtures of chemicals present at concentrations below or nearby their detection limits (Brooks et al. 2011).

The results obtained in these studies and their interpretation were comparable to those presently achieved. It is worth noting that IBR produces satisfactory discrimination between sites with different health status whatever the combination of biomarkers is. However, in order to avoid disparity of approaches and to give coherence to the biomarker approach two recommendations are made. Biomarkers should be selected at different levels of biological complexity and ordered accordingly, which facilitates comparisons and provides optimal information from star plots concerning the description of the biological responses to environmental changes/status. Secondly, a consensus number of biomarkers should be used or alternatively, as suggested by Broeg and Lehtonen (2006), IBR/n must be applied instead of IBR. Five biomarkers would be representative of the biological complexity levels from which biomarkers may provide information (molecular, cellular, tissue, individual, population).

#### Ecosystem health condition chart (EHCC)

EHCC was performed in order to describe environmental health condition of each locality on the basis of one exposure (AOX_exp_) and seven effect biomarkers (AOX_eff_, LP, Vv_L_, Vv_BAS_, MLR/MET, CI_IR_, and CI_PI_) according to each locality’s reference values (Marigómez et al. 2006; Orbea et al. 2006; Garmendia et al. 2010, 2011a, 2011b). EHCC showed a “bad ecosystem health condition” in most of the localities in 2003 and April-04. Signals of recovery towards “delicate” and “tolerable ecosystem health condition” conditions started sooner in Galicia but a “bad ecosystem health condition” persisted until October-05 in almost all the Bay of Biscay localities. Although some biomarkers return or nearly return to baseline values at different times from April-05 onwards, others such as AOX_exp_, MLR/MET, CI_IR_ and CI_PI_, remained “warning” and hence the “delicate ecosystem health” condition persisted in most localities until April-06.

This approach was previously used to integrate biomarker data in a multispecies ecotoxicological monitoring program carried out in 1993–1994 in the Reserve of the Biosphere of Urdaibai (Basque Coast in the Bay of Biscay) (RBU-Rep 1994; Díez 1996). *M.* *galloprovincialis*, *Crassostrea angulata*, *Hediste diversicolor*, *Carcinus maenas*, *Chelon labrosus*, *P. flexus*, *Potamochistus minutus* and *Chondrostoma polypeis* were used as sentinels in which different combinations of biological responses (Vv_L_, MLR/MET, digestive gland and liver histopathology, liver and gill parasitization, gonad development, flesh condition, spleen MMCs) and pollutant tissue-levels (organochemical compounds and metals) were measured in the different species. Then, EHCC was useful to identify “good ecosystem health condition” and those with “delicate” or “bad ecosystem health condition” (RBU-Rep 1994).

#### Integrative Biomarker Index (IBI)

IBI was designed including reference values (like BAI, HSI and EHCC), following the robust mathematical procedure used to compute IBR and representing star plots where biomarkers are ordered according to the levels of biological complexity (like BAI and HSI) (Beliaeff and Burgeot 2002; Broeg et al. 2005; Dagnino et al. 2007). The calculation of all the specific indices of biological response (MRI, CRI, TRI, SRI, and DRI) was designed in a way that the scale of this parameter should provide a easy output (“0” assigned to the reference status and “1” to the critical value). IBI was calculated by integrating MRI, CRI, TRI, SRI and DRI. Since the number of biomarkers is fixed (5 biological complexity levels), there is no need to calculate an average index such as IBR/n. Reference values are employed for its calculation. Thus, IBI can be used directly for comparison purposes, even among different sampling areas and times, and does not need to be recalculated when new data (samples, etc.) are introduced. Finally, any biomarker can be used as representative of each biological complexity level, provided that its biological mechanisms and its reference and critical values are sufficiently established.

According to the IBI, ecosystem health status was most affected in the first sampling year (April-03–April-04). Environmental condition worsened in April-05/July-05 but recovered again in October-05. In contrast, Laredo and Arrigunaga showed moderately high-to-high IBI values all along the study period, which might be related to the presence of chronic pollution.

Like in the case of IBR, star plots are used to provide complementary information concerning mechanisms of biological effects of contaminants. Star plots revealed details about the biological responses elicited at each sampling time and locality. Effects at the molecular and cellular levels (MRI and CRI) were first recorded (2003 and April-04), whereas TRI was eventually dominant in Galician localities (Oia, Aguiño, Caldebarcos, Camelle) in April-03, DRI in Suances in April-04, and SRI and DRI in Oia in April-06 and in Arrigunaga in April-05 and April-06. It seems therefore that after April-04, the responses at tissue and individual and population level gained relevance in Biscay Bay, particularly in Arrigunaga and Suances, but most biomarkers remained lowered and balanced in Galicia. Exceptionally, in S. Bartolomeu the molecular responses were dominant in April-05, and in Oia the biomarkers at the individual/population level were dominant in April-06. Thus, Arrigunaga might represent a chronically polluted site, whereas S. Bartolomeu and Oia may have eventually result environmentally distressed.

#### Comparison of ecosystem health indices

The main objective of the present investigation was to compare the different available indices for biomarker integration in order to determine each other’s advantages and disadvantages, and contribute to the existing debate about their convenience and reliability in an attempt to avoid an undesired proliferation of indices and to establish solid criteria for their selection depending on the user’s circumstances and capabilities.

BAI is a graded biomarker index, which allows statistical comparisons of toxically induced alterations among data sets obtained at different geographical areas (Broeg et al. 2005). BAI responds to a variety of pollutants and integrates their interactions, and has been demonstrated to link with alterations at the ecosystem level (Broeg et al. 2005; Broeg and Lehtonen 2006). BAI can be implemented in routine biomonitoring programs (Broeg and Lehtonen 2006), although it only provides a gross estimation of the degree of environmental condition, and can be only applied using biomarkers whose reference and critical values are previously known. Overall, its resolution is limited and does not provide information about the mechanisms of biological response.

The expert system requires a relatively good knowledge of the mechanisms underlying the development of the stress syndrome induced in mussels by pollutants (Dagnino et al. 2007). It uses biomarkers that are sensitive to stress at a molecular, cellular, tissue and organism levels and that are characterized by different stress-response profiles (Viarengo et al. 2007). Biomarkers characterized by increasing and decreasing trends, such as LP, may reveal the progression of the stress syndrome from early responses to pathological condition (Dagnino et al. 2007). Bell-shaped biomarkers respond transiently at early stages of the stress syndrome and LP is needed to correctly interpret them and compute HSI by the expert system (Dagnino et al. 2007). The output of the expert system presents a good resolution power to distinguish different degrees of environmental stress both in laboratory and field studies (Viarengo et al. 2000; Dondero et al. 2006; Dagnino et al. 2007, present results). However, unless measurements at the organism level (i.e. SOS and SFG) are employed, which in some circumstances might result difficult, the “pathological health status” cannot be identified. Overall, the main difficulties of the expert system are that (a) HSI is only computable when a reference site or an experimental control is available; and (b) a relatively good background knowledge of the biological responses involved is needed. In contrast, it has the advantages of (a) integrating different biological responses regarding their level, type and response profile, (b) providing a synthetic index; (c) being user-friendly for environmental managers; and (d) providing a cost-effective approach for “biological Mussel Watch” based on a two-tier approach (Dagnino et al. 2007; Viarengo et al. 2007). The two-tier approach consists of Tier 1 (screening using high sensitive low cost biomarkers such as LP) and Tier 2 (determining HSI after application of a suite of biomarkers with LP as guide parameter) (Viarengo et al. 2007). Thus, in those cases where Tier 1 is not responsive, there is no need to apply the expert system.

The IBR index succeeds in identifying temporal and spatial fluctuations in ecosystem health status and their magnitude and produces consistent results regardless of the combination of biomarkers used as a suite for its calculations (Broeg and Lehtonen 2006). However, due to the existence of a stress syndrome in spring-early summer, associated to the reproductive cycle (Leiniö and Lehtonen, 2005), seasonal comparisons are only possible when the biomarkers used in the IBR index calculations are known to be unaffected by season (Broeg and Lehtonen 2006). In addition, the successful application of the IBR depends on a priori choices of biomarkers and the number of them (Broeg and Lehtonen 2006). Even more, different IBR index values are obtained depending on the arrangement of the same biomarkers in the star plots. In order to solve this question, Broeg and Lehtonen (2006) calculated several IBR values for the same data, changing the order of biomarkers and using the mean of all the index values as the final IBR index.

Due to its mathematical basis, the IBR becomes more robust when the number of biomarkers increases (Broeg and Lehtonen 2006), the “relative weight” of each biomarker being markedly reduced when the set of biomarkers is relatively large (6–8 biomarkers) (Beliaeff and Burgeot 2002; Broeg and Lehtonen 2006; Damiens et al. 2007). However, we must consider that large suites of biomarkers would confer a more similar weight to every biomarker and not all of them involve equal environmental relevance (i.e. priority is given to LP in BAI, HSI and EHCC; Broeg et al. 2005; Dagnino et al. 2007). Moreover, IBR may also provide false negative results since IBR index calculations are based on the z-score approach. This index is biased and if one single biomarker value is “zero” the IBR index will be low regardless of whether the remainder biomarker values are high (Broeg and Lehtonen 2006). Finally, the IBR must be re-calculated every time that new biomarker, new site or new comparing season values are introduced in the data set (Broeg and Lehtonen 2006). Thus, new data must be incorporated and processed together with the previous ones, resulting in new IBR values. Broeg and Lehtonen (2006) described the IBR as a “dynamic” index that does not assign a fixed numerical value to a given ecosystem health status. Thus, it does not allow for direct inter-site and inter-time comparisons and the new data must be incorporated and processed together with the previous ones to obtain new comparable IBR values (Broeg and Lehtonen 2006). If all these withdrawn are taken into account (seasonal sampling, biomarker selection and order, etc.), the IBR can be very useful for biomonitoring in those geographical areas where reference values are not available, as well as for those biomarkers with not well established reference values, and also when biomarkers at complex levels of biological complexity are not available. Additionally, it provides indication of the biological mechanisms involved in environmental injury, which might serve for diagnostic purposes and can be related to the levels of specific pollutants (i.e. by comparing biomarker and pollutant star plots).

EHCC provides a user-friendly indication of the different levels of ecosystem health together with mechanistic information needed to characterize the stress syndrome. EHCC is based on compliance with eight biological responses covering exposure and effect biomarkers at different levels of biological complexity. The ecosystem health status is assigned according to some guideline criteria that involve sensitive general stress biomarkers such as LP and general criteria that define the range of response for a suite of biomarkers of different nature and biological complexity level. The strategy performed in order to obtain a classification of healthy status resembles that employed to award blue flags for beaches and marinas; the guideline criteria have to be fulfilled together with a minimum number of general criteria. If the guideline criteria or some of the general criteria are not fulfilled different degrees of stress syndrome are recognized. In order to perform the EHCC, there is a need to use (maybe season dependent) reference baseline values for the specific local area studied but, in contrast, direct data are used without any mathematical treatment and the incorporation of new data can be easily done. Thus, EHCC is useful for the surveillance of changes in the health status of particular protected/interesting areas (i.e. Natural parks, Biosphere reserves, etc.) and condensates information corresponding to studies carried out for long-term at large geographical areas in an easily understandable diagram that can be visually interpreted. Different color spots represent the degree of response of each particular biomarker, whereas the background color is a direct indication of the health status of a site at a particular moment. Thus, the informative value and drawbacks are similar to those discussed for BAI but the output is user-friendly (like the traffic light code used in BRI, a derivative of BAI; Hagger et al. 2008) and does not depend on mathematics, which might do it more attractive for non-scientific users. It was useful in its first application to monitor health status in the Urdaibai estuary in 1993–1994 (RBU-Rep 1994; Díez 1996) and it has been satisfactorily applied again to obtain an integrated view of the POS effects. Presently, the name of the categories according to RBU-Rep (1994) has been changed to adapt to those that represent varying degrees of severity from normal reference responses, as recommended under the WFD for ecological and chemical parameters (Environmental Agency 2002) and for BRI (Hagger et al. 2008).

IBI succeeds in identifying temporal and spatial fluctuations in ecosystem health status and their magnitude and may produce consistent results regardless of the combination of biomarkers used as a suite for its calculations. Seasonal comparisons are possible since the corresponding reference values for the biomarkers used are known. The successful application of IBI does not depend on a priori choices of biomarkers and the number of them: it has been fixed that five biomarkers corresponding to five levels of biological complexity (MRI, CRI, TRI, SRI, and DRI) must be used ordered from simple to complex levels of biological complexity. However, the biomarker representative of each biological complexity might change depending on the user’s circumstances and capabilities. The IBI calculations are not based on the z-score approach and therefore do not present the calculation difficulties reported for IBR (Broeg and Lehtonen 2006). However, individual biomarkers need a mathematical transformation to fix “0” as the reference biological response value and “1” as the critical value, which requires good background knowledge of the biomarker mechanisms and baseline values and variability in the study area, which is not always possible. Thus, the IBI value increases with damage to the ecosystem health status, which may allow for inter-sites and inter-times comparisons. High IBI values may result from the warning sign of a single biological response (>1) or by summing-up less marked biological responses. IBI is very useful for biomonitoring in those geographical areas where reference values are available, as well as for those biomarkers with well established reference values (i.e. long-term monitoring of the POS biological effects). Additionally, like IBR, it provides indication of the biological mechanisms involved in environmental injury, and like BAI, HSI and EHCC, considers different levels of biological complexity from molecular (MRI) to population (DRI), linking molecular responses with alterations potentially relevant at the ecosystem level (Broeg et al. 2005; Dagnino et al. 2007; Viarengo et al. 2007).

## Conclusions

Overall concordance was observed among the five indices, which revealed a severe environmental stress in 2003 and in April-04 and a trend of recovery after July-04. In previous studies, successful results were also obtained when comparing different indices such as BAI, IBR and HSI (Broeg and Lehtonen 2006; Dagnino et al. 2007). As a general rule, all these integrative indices provide comprehensive information about the degree of biological effects of pollution in marine organisms and may therefore serve as a useful tool for environmental managers. However, the information provided by each index may be substantially different (Table 5). Thus, for instance, whereas strong impact in 2003 and recovery in 2006 is evidenced by HSI for most localities (Fig. 3), BAI, which is less sensitive, reveals less pronounced initial impact and incomplete recovery in 2006 (Fig. 2); with EHCC in between, sensitive to the initial impact for over 1 year but not fully recovered in 2006 (Fig. 6). Indeed, future research efforts should be addressed to achieve a proper calibration between the different indices.

**Table 5:** Comparison of the five different integrative indices of ecosystem health status

Indices	Control values necessary	Type of data	Relevance to environmental health status	Sensitivity	Biomarkers knowledge
BAI	No	Effect biomarkers	Simple indication	General screening	Yes
ES	Yes	Effect/exposure biomarkers	Simple indication	Conclusive approach	Yes
IBR	No	Effect/exposure biomarkers	Mechanistic information	Conclusive approach	No
IBI	No	Effect/exposure biomarkers	Mechanistic information	Conclusive approach	Yes
EHCC	No	Effect/exposure biomarkers	Mechanistic information	Conclusive approach	Yes

First, the selection of biomarkers is a crucial issue. BAI and IBI are only based on biomarkers of general stress while HSI, IBR and EHCC can be constructed using both effect and exposure biomarkers.

Second, whereas BAI, HSI, IBI and EHCC require a more or less extensive knowledge of mechanisms of the biological response and the existence of reference/critical values, IBR is a simple mathematical transformation which does not need such knowledge. On the other hand, EHCC allows describing each scenario using pure biomarkers without any kind of transformation. A better knowledge of reference and critical values and the natural variability of biomarkers, both at global and local scales, will enhance the power and reliability of BAI, HSI, IBI and EHCC. Meanwhile, in the present study, statistical approaches commonly used in epidemiological studies (using median and mode values as discriminating parameters) have been successfully applied for IRI and PII, which refer to immune and disease condition. Although their discriminating power will benefit from the establishment of actual baseline/reference data at local scale, which are hence urgently needed, it is conceivable that any deviation from “normality” even in absence of baseline data is symptomatic, as shown herein.

Third, whereas BAI and HSI provide a basic indication of the ecosystem health status, IBR, IBI and EHCC provide complementary information concerning the mechanisms of biological response to environmental insult. Particularly, IBR and IBI accompanying start plots and the EHCC color spots depicted within each colored background framework are very useful for this purpose. Stakeholders that want to know more details can, through these means, get the elaborated information of the individual biomarkers, not as inaccessible raw data but as relative to baseline and critical values in the context of the study case.

Consequently, any of these indices may be valuable for an oil-spill event. The selection of the indices and the biomarkers used for their calculation depends on (a) the researchers’ expertise and technical capability as regards biomarkers; (b) the existence of reference/critical values or previous studies in the impacted area; and (c) the available resources. In the circumstances of the present study, EHCC, IBR and IBI provide the most precise information about the POS biological consequences.

Overall, the use of integrative indices describing pollution-induced stress constitutes a useful tool for environmental managers and scientists. However, due to either their intrinsic characteristics or to their still limited degree of development, the results they provide cannot be taken at present as “face value” but rather as tools to direct further actions in the attempt to resolve causes of the differences observed in ecosystem health status, as previously stated by Broeg and Lehtonen (2006). Hopefully, the more we use them under this viewpoint, the less dubious and more powerful they will become; thus, a set of them could be, in the near future, as widely accepted and useful as the market indices in today’s economy.

## Abbreviations

AChE: Acetylcholinesterase activity
ADDU: DNA adducts
AOX: Acyl-CoA-oxidase activity
BAI: Bioeffect Assessment Index
CAT: Catalase activity
CI: Cumulative intensity
CI_IR_: Cumulative intensity of inflammatory responses
CI_PI_: Cumulative intensity of parasitization
CRI: (Sub)cellular Response Index
DRI: Disease Response Index
ECHH: Ecosystem health condition chart
ERI: Epithelial Response Index
EROD: Ethoxyresorufin-*O*-deethylase
GST: Glutathione-*S*-transferase activity
HSI: Health Status Index
IBI: Integrative biomarker index
IBR: Integrated biological response
IRI: Inflammatory response index
LMS: Lysosomal membrane stability
LP: Labilization period of lysosomal membrane
LPF: Accumulation of lipofuscins
LRI: Lysosomal Response Index
MAD: Malonyl dialdehyde
MET: Mean epithelial thickness (digestive gland epithelium)
MLR: Mean luminal radius (digestive gland epithelium)
MMCs: Melanomacrophage centers
MN: Induction of micronuclei
MRI: Molecular/Metabolic Response Index
MT: Methallothioneins
NL: Intracellular accumulation of neutral lipids
PII: Parasitic Infestation Index
POS: Prestige oil spill
SFG: Scope-for-growth
SOS: Stress-on-stress
SRI: Systemic Response Index
S/V_L_: Lysosomal surface-to-volume ratio
TBARS: Thiobarbituric acid reactive substances
TRI: Tissue Response Index
Vv_BAS_: Volume density of basophilic cells
Vv_L_: Lysosomal volume density
